# Estimating the morbidity and mortality associated with infections due to multidrug-resistant bacteria (MDRB), France, 2012

**DOI:** 10.1186/s13756-016-0154-z

**Published:** 2016-12-12

**Authors:** M. Colomb-Cotinat, J. Lacoste, C. Brun-Buisson, V. Jarlier, B. Coignard, S. Vaux

**Affiliations:** 1 Santé Publique France, The French Public Health Agency, F-94415 Saint-Maurice, France; 2 Assistance publique-hôpitaux de Paris, CHU Henri Mondor, F-94000 Créteil, France; 3 Sorbonne Universités, UPMC Univ Paris 06, Inserm, Centre d’Immunologie et des Maladies Infectieuses, UMR 1135 & APHP, CHU Pitié-Salpêtrière, Laboratoire de Bactériologie-Hygiène, F-75013 Paris, France

**Keywords:** Antimicrobial resistance, Epidemiology, Morbidity, Mortality, Infection due to multidrug resistant bacteria, France

## Abstract

**Background:**

A study based on 2007 data estimated that 386,000 infections due to multidrug-resistant bacteria (MDRB) occurred in Europe that year and 25,000 patients died from these infections. Our objective was to estimate the morbidity and mortality associated with these infections in France.

**Methods:**

The MDRB considered were methicillin-resistant *Staphylococcus aureus* (MRSA), glycopeptide-resistant *enterococci*, third-generation cephalosporin-resistant (3GC-R) *Escherichia coli* and *Klebsiella pneumoniae*, carbapenem-resistant *Klebsiella pneumoniae*, *Acinetobacter* spp. and *Pseudomonas aeruginosa* (CR *P. aeruginosa*). The number of invasive infections (infections with bacteria isolated from blood or cerebrospinal fluid) due to MDRB, as reported by France to EARS-Net in 2012, was corrected for the coverage of our surveillance network and extrapolated to other body sites using ratios from the French healthcare-associated infections point prevalence survey and the literature. Mortality associated with MDRB infection was estimated using proportions from the literature. Methods and parameters were reviewed by a panel of experts.

**Results:**

We estimate that 158,000 (127,000 to 245,000) infections due to MDRB occurred in 2012 in France (incidence: 1.48 to 2.85 per 1000 hospital days), including 16,000 invasive infections. MRSA, 3GC-R *E. coli* and *K. pneumoniae* were responsible for 120,000 (90,000 to 172,000) infections, i.e., 75% of the total. An estimated 12,500 (11,500 to 17,500) deaths were associated with these infections, including 2,700 associated with invasive infections. MRSA, 3GC-R *E. coli* and CR *P. aeruginosa* accounted for 88% of these deaths.

**Conclusion:**

These first estimates confirm that MRSA, 3GC-R *Escherichia coli* and *Klebsiella pneumoniae* account for the largest portion of the morbidity and mortality of infections due to MDRB in France. These results are not directly comparable with the European study because the methodology used differs in many respects. The differences identified between our study and previous studies underline the need to define a standardised protocol for international assessments of the morbidity and mortality of antibiotic resistance. Estimating morbidity and mortality will facilitate communication and awareness in order to reinforce adherence and support of healthcare professionals and policy-makers to MDRB prevention programs.

**Electronic supplementary material:**

The online version of this article (doi:10.1186/s13756-016-0154-z) contains supplementary material, which is available to authorized users.

## Background

Antibiotic resistance is a constantly evolving phenomenon and a threat to infection and disease control; it complicates patient management and treatment strategy and prolongs hospital stays. Nowadays, this international public health problem is recognised as one of the scourges of the 21st century [1].

Several studies have sought to estimate the morbidity and mortality of infections due to multidrug-resistant bacteria (MDRB). A joint report from the European Centre for Disease Prevention and Control (ECDC) and the European Medecines Agency (EMEA), published in 2009 and based on data from 2007 [2], estimated at approximately 386,000 the annual number of infections due to MDRB in Europe that year, including 42,500 cases (11%) of bloodstream infections. The number of deaths associated with these infections was estimated at more than 25,000. A report by the US Centers for Disease Control and Prevention (CDC) from 2013 [3] provided an overview of the annual morbidity and mortality of antibiotic-resistant infections in the United States, estimating their number at approximately 2 million and the number of deaths associated with these infections at 23,000. These two studies, although they used different methods and did not consider the same panel of microorganisms, both underlined the important morbidity and mortality of antibiotic resistance on public health.

No corresponding estimate has been so far available for France, despite its wealth of antibiotic-resistance surveillance networks. These networks focus on specific pathogens and sometimes on specific sources of samples [4, 5]. The data they provide are useful for assessing trends and detecting epidemics. Nonetheless, they do not provide an overall view of the MDRB morbidity and mortality nor do they permit simple communication on this topic.

The objective of this study conducted by Santé publique France (the French national public health agency) was to estimate for the first time the morbidity and mortality (number of cases and number of attributable deaths) of infections due to MDRB in France, in order to advocate for strategies to control and prevent them, to guide public health authorities in implementing these policies, and to support communication towards healthcare professionals and the general public.

## Methods

### Microorganisms selected

The MDRB considered in this study were defined using the following criteria:

- being associated with invasive infections, i.e., infections with a bacteria isolated from blood or cerebrospinal fluid [5];
- having a significant prevalence or having emerged recently;
- being included in a surveillance network in France;
- being multidrug-resistant (MDR) as defined in the European Antimicrobial Resistance Surveillance Network (EARS-Net) protocol [5].

We thus selected eight bacteria–antibiotic combinations:

- *Staphylococcus aureus* resistant to methicillin (MRSA);
- *Enterococcus faecium* and *E. faecalis* resistant to glycopeptides (GRE);
- *Escherichia coli* resistant to third-generation cephalosporins (3GC-R *E. Coli*);
- *Klebsiella pneumoniae* resistant to third-generation cephalosporins (3GC-R *K. pneumoniae*);
- *Pseudomonas aeruginosa* resistant to carbapenems (CR *P. aeruginosa*);
- *Klebsiella pneumoniae* resistant to carbapenems (CR *K. pneumoniae*);
- *Acinetobacter spp.* resistant to carbapenems (CR *Acinetobacter spp*).

According to the EARS-Net protocol, antibiotics considered for resistance were a) for MRSA: oxacillin, methicillin, flucloxacillin, cloxacillin, dicloxacillin and cefoxitin (and PCR mecA or PBP2a detection); b) for GRE: vancomycin; c) for 3GC-R *E.coli* and 3GC-R *K. pneumoniae*: cefotaxime, ceftriaxone, ceftazidime; d) for CR *P. aeruginosa*; imipenem, meropenem; e) for CR *K. pneumoniae*: imipenem, meropenem; f) for CR *Acinetobacter spp*: imipenem, meropenem, doripenem.

*Streptococcus pneumoniae* with reduced penicillin susceptibility was not considered as MDRB in accordance with generally accepted microbiological criteria [6].

### *Number of infections* due to MDRB

To select the most frequently diagnosed infectious body sites for each of these MDRB, data from the 2012 point prevalence survey on healthcare-associated infections and antimicrobial use in French hospitals (2012 PPS) [7] were used. This survey, conducted every five years on a single day in nearly all French healthcare facilities collects standardised data about nosocomial infections. In 2012, data were collected for 91% of French hospital beds.

Based on this data, the following infectious body sites were selected, as there were the most frequent infections in the 2012 PPS:

- for all MDRB: invasive infections (bacteria isolated from blood or cerebrospinal fluid), urinary tract infections, skin and soft tissue infections, and surgical site infections;
- for all MDRB excluding GRE: respiratory tract infections;
- for MRSA only: bone and joint infections (does not include surgical site infections);
- for GRE only: gastrointestinal tract infections (gastro-intestinal and intra-abdominal infections).

The number of invasive infections for each MDRB was estimated from data transmitted by France to EARS-Net [5]. This European network collects resistance data of bacterial strains isolated from invasive infections (bacteria isolated from blood or cerebrospinal fluid). As duplicate isolates for the same patients had already been eliminated during data collection, the number of incident cases of invasive infections due to MDRB occurring each year in the network was directly estimated from the number of resistant strains isolated. For instance, there were 1005 strains of MRSA reported in EARS-Net for France in 2012, so the number of incident cases of invasive infections due to MRSA in 2012 in the French EARS network was estimated as 1005.

The number of incident cases of invasive infections was then corrected by taking into account the coverage of the EARS-Net network for France, estimated from the number of inpatient hospital days (HD) reported by hospitals whose laboratories had transmitted data to the network (secondary and tertiary care hospitals). The list of these hospitals was obtained from the Epibac network [8] and the total number of HD from the 2012 Health Facilities Annual Statistics (SAE) report [9]. The EARS-Net coverage was estimated by dividing the number of HD in the participating hospitals (N_1_) by the total number of HD in all French secondary and tertiary care hospitals that same year provided by the same source (N_2_). For instance, the number of cases of invasive infections due to MRSA in 2012 for France was estimated as 5574 (n_1_). The incidence of patients with an invasive infection was also expressed as the number of cases per 1000 HD.

In order to estimate the number of cases with infections at other body sites, the ratio between the number of cases with infections at a given body site and the number of cases of invasive infections was calculated for each MDRB and each body site using data from the 2012 PPS. For instance, to evaluate the number of cases of urinary tract infections (UTI) due to MRSA in our study, we first calculated a specific ratio between the number of UTI due to MRSA reported in the 2012 PPS (*n* = 103) divided by the number of invasive infections due to MRSA reported in the 2012 PPS (*n* = 105). The specific ratio for UTI due to MRSA was 103/105 = 0.98. We then multiplied the number of cases with invasive infections due to MRSA estimated in our study based on EARS-Net data (n_1_ = 5574) by this ratio (0.98), to estimate the number of cases with urinary tract infections due to MRSA in France (n_11_ = 5468). The same approach was used for other body sites and MDRB.

In order to have intervals of plausibility around these estimates, we also estimated these ratios from a non-systematic review of the literature targeting French and European publications when available. Plausibility intervals were then calculated using high and low ratios estimated from the literature. Table 1 presents the ratios that were used, for each MDRB and body sites, to estimate the incidence of cases of non-invasive infections; ratios calculated from the French PPS 2012 were used as target values, and ratios excerpted from the literature as low and high values for intervals of plausibility.

**Table 1:** Ratios used to estimate the incidence of cases of non-invasive infections, France 2012

Body sites	PPS ratio	Ratios from the literature	
		Low value	High value
No. MRSA urinary tract infections/No. MRSA invasive infections	0.98 [7]	0.75 [11]	2.30 [12]
No. MRSA respiratory infections/No. MRSA invasive infections	2.37 [7]	1.25 [11]	2.50 [12]
No. MRSA skin and soft tissue infections and surgical site infections/No. MRSA invasive infections	4.17 [7]	4.90 [12]	5.25 [11]
No. MRSA bone and joint infections/No. MRSA invasive infections	0.79 [7]	^b^	1.38 [12]
No. GRE urinary tract infections/No. GRE invasive infections	4.33 [7]	2.33 [13]	3.44 [14]
No. GRE gastrointestinal tract infections /No. GRE invasive infections	2.65 [7]	1.89 [14]	^b^
No. GRE skin and soft tissue infections and surgical site infections/No. GRE invasive infections	1.46 [7]	4.67 [14]	5.00 [13]
No. 3GC-R *E. coli* urinary tract infections/No. 3GC-R *E. coli* invasive infections	5.50 [7]	2.30 [13]	^b^
No. 3GC-R *E. coli* respiratory tract infections/No. 3GC-R *E. coli* invasive infections	1.08 [7]	^b^	2.50 [13]
No. 3GC-R *E. coli* skin and soft tissue infections and surgical site infections/No. 3GC-R *E. coli* invasive infections	1.37 [7]	^b^	4.90 [13]
No. 3GC-R *K. pneumoniae* urinary tract infections/No. 3GC-R *K. pneumoniae* invasive infections	2.90 [7]	1.19 [15]	2.30 [13]
No. 3GC-R *K. pneumoniae* respiratory tract infections/No. 3GC-R *K. pneumoniae* invasive infections	2.55 [7]	1.19 [15]	2.50 [13]
No. 3GC-R *K. pneumoniae* skin and soft tissue infections and surgical site infections/No. 3GC-R *K. pneumoniae* invasive infections	0.82 [7]	0.33 [15]	4.90 [13]
No. CR *K. pneumoniae* urinary tract infections/No. CR *K. pneumoniae* invasive infections	2.71 [7]	3.00 [16]	3.10 [17]
No. CR *K. pneumoniae* respiratory tract infections/No. CR *K. pneumoniae* invasive infections	3.10 [7]	0.42 [17]	1.60 [16]
No. CR *K. pneumoniae* skin and soft tissue infections and surgical site infections/No. CR *K. pneumoniae* invasive infections	0.44^a^	0.28 [17]	0.60 [16]
No. CR *P. aeruginosa* urinary tract infections/No. CR *P. aeruginosa* invasive infections	2.11 [7]	^b^	11.30 [15]
No. CR *P. aeruginosa* respiratory tract infections/No. CR *P. aeruginosa* invasive infections	10.99 [7]	^b^	16.00 [15]
No. CR *P. aeruginosa* skin and soft tissue infections and surgical site infections/No. CR *P. aeruginosa* invasive infections	3.07 [7]	^b^	4.67 [15]
No. CR *Acinetobacter* urinary tract infections/No. CR *Acinetobacter* invasive infections	0.58^a^	0.32 [18]	0.83 [17]
No. CR *Acinetobacter* respiratory tract infections/No. CR *Acinetobacter* invasive infections	4.64 [7]	1.21 [18]	3.83 [17]
No. CR *Acinetobacter* skin and soft tissue infections and surgical site infections/No. CR *Acinetobacter* invasive infections	0.73^a^	0.29 [18]	1.17 [17]

^a^no 2012 PPS data for these infections because the prevalence of these MDRB is too low in France; the ratio value applied is the mean value of the 2 ratios from the literature

^b^no data in the literature for these infections; the value of the 2012 PPS ratio was applied

The total number of infections due to MDRB was finally calculated by adding the number of infections obtained for each MDRB species and each body site considered.

### Number of deaths associated with infections due to MDRB

For each infection due to MDRB and body site considered, the number of deaths was estimated by applying to the number of cases of infections estimated above the proportion of deaths associated with infection due to MDRB, based on a non-systematic review of the literature (Table 2) that focused on publications reporting proportions of deaths associated with infections due to MDRB considered. Results from French or European studies were used preferentially when available. The mortality indicator used in our study was therefore the number of deaths associated with MDRB infections as a whole, but not the number specifically associated with antibiotic resistance per se. Consequently, this study did not estimate the extra deaths relative to infections due to antibiotic-susceptible bacteria.

**Table 2:** Proportion of mortality associated with infections due to MDRB, France 2012

Body sites	Associated deaths (%)	Reference	Year of publication
MRSA invasive infections	9.8	[19]	2003
MRSA urinary tract infections	0.2	[10, 19]	1998
MRSA respiratory tract infections	7.0	[10, 19]	1998
MRSA skin and soft tissue infections and surgical site infections	1.4	[10, 19]	1998
MRSA bone and joint infections	9.8	^d^	
GRE invasive infections	25.0	[14]	2002
GRE urinary tract infections	9.0	[14]	2002
GRE gastrointestinal tract infections	3.0	[14]	2002
GRE skin and soft tissue infections and surgical site infections	6.0	[14]	2002
3GC-R *E. coli* invasive infections	18.0	[20]	2010
3GC-R *E. coli* urinary tract infections	0.0	[21]	2006
3GC-R *E. coli* respiratory tract infections	12.9	[10, 20]	1998
3GC-R *E. coli* skin and soft tissue infections and surgical site infections	2.6	[10, 20]	1998
3GC-R *K. pneumoniae* invasive infections	18.0	[20]	2010
3GC-R *K. pneumoniae* urinary tract infections	0.4	[10, 20]	1998
3GC-R *K. pneumoniae* respiratory tract infections	12.9	[10, 20]	1998
3GC-R *K. pneumoniae* skin and soft tissue infections and surgical site infections	2.6	[10, 20]	1998
CR *K. pneumoniae* invasive infections	37.0	[22]	2010
CR *K. pneumoniae* urinary tract infections	0.8	[10, 22]	1998
CR *K. pneumoniae* respiratory tract infections	26.4	[10, 22]	1998
3GC-R *K. pneumoniae* skin and soft tissue infections and surgical site infections	5.4	[10, 22]	1998
CR *P. aeruginosa* invasive infections	33.0	[23]	2010
CR *P. aeruginosa* urinary tract infections	0.8	[10, 23]	1998
CR *P. aeruginosa* respiratory infections	23.6	[10, 23]	1998
CR *P. aeruginosa* skin and soft tissue infections and surgical site infections	4.8	[10, 23]	1998
CR *Acinetobacter* invasive infections	36.5	[24]	2007
CR *Acinetobacter* urinary tract infections	0.8	[10, 24]	1998
CR *Acinetobacter* respiratory infections	26.1	[10, 24]	1998
CR *Acinetobacter* skin and soft tissue infections and surgical site infections	5.3	[10, 24]	1998

^d^no data could be found in literature, the fraction applied is therefore that estimated for invasive infections. Invasive infections: bacteria isolated from blood or cerebrospinal fluid

When a value for the associated mortality for a body site could not be found in the literature, it was estimated from the mortality associated with invasive infections (mainly bloodstream infections) for each of the MDRB studied, applying a correction factor from a US publication [10].

The total number of deaths associated with an infection due to MDRB was calculated by adding the number of associated deaths obtained for each MDRB and each body site studied.

We jointly validated the choices of the different parameters used in this study (ratios used to estimate the incidence of cases of non-invasive infections and associated mortality proportion) after a critical review with external experts, clinicians and microbiologists.

**Fig. 1:**
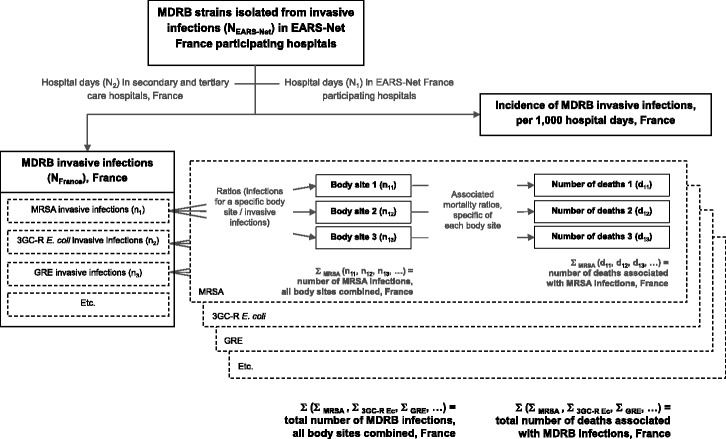
Methodology for estimating the morbidity and mortality of infections due to MDRB in France, 2012

Figure 1 summarises the methodology used to estimate morbidity and mortality associated with infections due to MDRB in France.

## Results

### Estimate of EARS-Net coverage, France

We estimate that in 2012 EARS-Net covered 18% of HD in secondary and tertiary care hospitals in France.

### Number of infections due to MDRB

The number of infections due to MDRB was estimated at approximately 158,000 in France in 2012, for an incidence of 1.83 per 1,000 HD. Data from the literature enabled us to estimate a plausibility interval for the annual number of cases from 127,000 to 245,000 (incidence: 1.48 to 2.85 per 1,000 HD).

Most cases were infections caused by MRSA (33%; plausibility interval: 28 to 38%), 3GC-R *E. coli* (32%; 26 to 32%), CR *P. aeruginosa* (23%; 23 to 29%) or 3GC-R *K. pneumoniae* (10%; 6 to 10%). The other infections due to MDRB considered accounted for less than 1% of all cases. Most infections were due to Gram-negative bacteria (67%; 62 to 71%).

Table 3 details the number and incidence rates of infections according to whether or not the infection was invasive. The total number of invasive infections due to MDRB was estimated at approximately 16,000 in 2012 (incidence: 0.185 per 1,000 HD), or 10% (6 to 13%) of all MDRB infections. Overall, 70% of the invasive infections were due to MRSA or 3GC-R *E. coli.*

**Table 3:** Annual number and incidence rate of infections due to MDRB, France 2012

MDRB	Body site	Total number of cases (%)						Incidence per 1,000 HD		
		PPS ratio		Ratios from literature				PPS ratio	Ratios from literature	
				Low value		High value			Low value	High value
MRSA	Invasive inf.	5,574	(10.8%)	5,574	(11.5%)	5,574	(8.0%)	0.065	0.065	0.065
	Others	46,270	(89.2%)	42,852	(88.5%)	63,710	(92.0%)	0.538	0.498	0.741
	Total	51,844	(100%)	48,426	(100%)	69,284	(100%)	0.603	0.563	0.806
GRE	Invasive inf.	39	(10.6%)	39	(10.1%)	39	(8.3%)	<0.001	<0.001	<0.001
	Others	328	(89.4%)	345	(89.9%)	430	(91.7%)	0.004	0.004	0.005
	Total	367	(100%)	384	(100%)	469	(100%)	0,004	0,004	0,005
3GC-R *E. coli.*	Invasive inf.	5,690	(11.2%)	5,690	(17.4%)	5,690	(7.2%)	0.066	0.066	0.066
	Others	45,226	(88.8%)	27,028	(82.6%)	73,395	(92.8%)	0.526	0.314	0.854
	Total	50,916	(100%)	32,719	(100%)	79,085	(100%)	0.592	0.381	0.920
3GC-R *K. pneumoniae*	Invasive inf.	2,246	(13.8%)	2,246	(27.0%)	2,246	(9.3%)	0.026	0.026	0.026
	Others	14,068	(86.2%)	6,087	(73.0%)	21,789	(90.7%)	0.164	0.071	0.253
	Total	16,314	(100%)	8,333	(100%)	24,035	(100%)	0,190	0.097	0.280
CR *K. pneumoniae*	Invasive inf.	94	(13.8%)	94	(21.3%)	94	(15.9%)	0.001	0.001	0.001
	Others	589	(86.2%)	349	(78.7%)	500	(84.1%)	0.007	0.004	0.006
	Total	683	(100%)	443	(100%)	594	(100%)	0.008	0.005	0.007
CR *P. aeruginosa*	Invasive inf.	2,141	(5.8%)	2,141	(5.8%)	2,141	(3.0%)	0.025	0.025	0.025
	Others	34,616	(94.2%)	34,616	(94.2%)	68,442	(97.0%)	0.403	0.403	0.796
	Total	36,757	(100%)	36,757	(100%)	70,583	(100%)	0.428	0.428	0.821
CR *Acinetobacter spp*	Invasive inf.	111	(14.4%)	111	(35.5%)	111	(14.6%)	0.001	0.001	0.001
	Others	660	(85.6%)	202	(64.5%)	647	(85.4%)	0.008	0.003	0.008
	Total	771	(100%)	313	(100%)	758	(100%)	0.009	0.004	0.009
Total invasive inf.		15,895	(10.1%)	15,895	(12.5%)	15,895	(6.5%)	0.185	0.185	0.185
TOTAL		157,652	(100%)	127,375	(100%)	244,808	(100%)	1.834	1.481	2.847

Invasive infections: bacteria isolated from blood or cerebrospinal fluid

Infections caused by MRSA were mostly skin and soft tissue and surgical site infections, which altogether accounted for 56% (42 to 45%) of MRSA infections, with an incidence of 0.318 (0.270 to 0.340) per 1,000 HD. Urinary tract infections accounted for most of the 3GC-R *E. coli* infections (40%; range 40 to 61%), with an incidence of 0.152 (0.364-0.364) per 1,000 HD, and respiratory tract infections for most of the CR *P. aeruginosa* infections (64%; range 48 to 64%), with an incidence of 0.274 (0.274 to 0.398) per 1,000 HD.

### Number of deaths associated with infections due to MDRB

The number of deaths associated with infections due to MDRB in 2012 was estimated at 12,411 (11,422 to 17,470), of which approximately one fourth (2,800) were due to invasive infections. The corresponding incidence rate was estimated at 0.144 death per 1,000 HD (0.133 to 0.203).

More than half the deaths were associated with CR *P. aeruginosa* (53%; 54 to 58%) (Table 4). Infections caused by MRSA and by 3GC-R *E.coli* were responsible for respectively 16% (15 to 18%) and 18% (16 to 20%) of deaths. Overall, Gram-negative bacteria infections accounted for 83% (82 to 84%) of the estimated total deaths.

**Table 4:** Number of deaths associated with infections due to MDRB, France 2012

MDRB	Number of deaths associated with MDRB						Incidence per 1,000 HD		
	PPS ratios		Ratios from literature				PPS ratios	Ratios from literature	
			Low value		High value			Low value	High value
Gram +									
MRSA	2,236	(18.0%)	1,855	(16.2%)	2,711	(15.5%)	0.026	0.022	0.032
GRE	31	(0.3%)	31	(0.3%)	36	(0.2%)	<0.001	<0.001	<0.001
*Subtotal Gram +*	2,268	(18.3%)	1,886	(16.5%)	2,747	(15.7%)	0.026	0.022	0.032
Gram -									
3GC-R *E. coli.*	2,020	(16.3%)	2,020	(17.7%)	3,584	(20.5%)	0.023	0.023	0.042
3GC-R *K. pneumoniae*	1,217	(9.8%)	779	(6.8%)	1,436	(8.2%)	0.014	0.009	0.017
CR *K. pneumoniae*	116	(0.9%)	49	(0.4%)	80	(0.5%)	0.001	0.001	0.001
CR *P. aeruginosa*	6,610	(53.3%)	6,610	(57.9%)	9,464	(54.2%)	0.077	0.077	0.110
CR *Acinetobacter spp*	180	(1.4%)	78	(0.7%)	159	(0.9%)	0.002	0.001	0.002
*Subtotal Gram -*	10,143	(81.7%)	9,536	(83.5%)	14,723	(84.3%)	0.118	0.111	0.171
TOTAL	12,411	(100%)	11,422	(100%)	17,470	(100%)	0.144	0.133	0.203

Among the deaths associated with infections due to MDRB, 22% (16 to 24%) were due to invasive infections. This proportion was 11% (7 to 11%) for the CR *P. aeruginosa* infections, 24% (20 to 29%) for MRSA infections, and 51% (29 to 51%) for the 3GC-R *E. coli* infections.

## Discussion

Our study quantifies for the first time the morbidity and mortality of infections due to MDRB in France. We thus estimate at approximately 158,000 (127,000 to 245,000) the number of infections due to MDRB occurring in 2012 in France, including nearly 16,000 (6 to 12%) invasive infections (bloodstream infections and meningitis), MRSA and the two major *enterobacteriaceae* species resistant to third-generation cephalosporins account for three quarters (70 to 75%) of the infections recorded. The annual number of deaths associated with these infections is estimated at 12,500 (11,500 to 17,500), including 2,800 (22%) due to invasive infections; MRSA, 3GC-R *E. coli* and CR *P. aeruginosa* account for 88% (90 to 92%) of these deaths. Most of the infections considered in this study are healthcare associated infections, and 20 to 30% of them can be considered avoidable [25].

These results underestimate the morbidity and mortality of infections due to antibiotic-resistant bacteria in France. Although our study covers a large panel of MDRB and the most frequent body sites, it does not include all bacteria resistant to antibiotics and all infection sites. For example, as only the most frequent infections in the 2012 PPS data were selected for each MDRB, gastro-intestinal tract infections due to *Enterobacteriacae* were not taken into account in our study.

We did not take into account patients with more than one MDRB. However, this bias in underestimating MDRB infections should be very limited. Indeed, according to data from the 2012 French PPS, only 39 patients among a total of 15,180 infected patients (0.2%) had two or more different MDRB (data not shown).

Furthermore, the extrapolation of EARS-Net data to France was calculated on the number of inpatient hospital days reported only by French secondary and tertiary care hospitals, as the laboratories participating in the EARS-Net network are located only in such institutions. This may have underestimated the incidence of MDRB infections, as cases occurring in other types of hospitals have being ignored. Conversely, taking into account all healthcare institutions in France would have greatly overestimated the incidence since it is based on the assumption that the burden of the antibiotic resistance is comparable in all institutions.

The estimates produced in this study are consistent with those from other French data sources, especially from the BMR-RAISIN network [4], where between 4,000 and 5,000 cases of MRSA bloodstream infections and 4,000 to 9,000 cases of bloodstream infections due to extended-spectrum beta-lactamase (ESBL)-producing *enterobacteriaceae* — mostly with *E. coli* and *K. pneumoniae* — were estimated to occur in France in 2013.

The results of our study should also be examined in light of previous studies published in other countries. Nonetheless they are not directly comparable because the methodology used differs in many respects.

The European study [2] estimated there were more than 386,000 annual infections due to MDRB in Europe, and attributed 25,000 extra deaths to them. The EARS-Net data used for that European estimate date back to 2007, while the French study used more recent data (2012). Trends in the epidemiology of MDRB between 2007 and 2012, especially major changes for MRSA and 3GC-R enterobacteriaceae [5], may thus explain some of these differences. Indeed, Ears-Net data show the spread of 3GC-R resistance in *E. coli*, with a four-fold increase from 2.5% in 2007 to 10% in 2012.

In addition, the European study sought to examine the morbidity and mortality of MDRB associated with hospital-acquired infections only. This study considered only a fraction of all bloodstream infections, by correcting their incidence reported by EARS-Net by a factor derived from national prevalence studies to take into account only those of nosocomial origin, e.g., only 65.6% of MRSA bloodstream infections and 58.9% of 3GC-R *E. coli* bloodstream infections (ECDC, unpublished data). Moreover, the panels of infections and MDRB species considered in the present study were more complete than those of the European study, which did not include three MDRB from our panel (CR *K. pneumoniae*, *Acinetobacter spp*. and GR *Enterococcus faecalis*) and one infection site (bone and joint infections for MRSA).

Another major difference between the two studies is related to the correction of the EARS-Net data to estimate the number of cases for the entire country. The European study estimated the incidence of invasive infections due to resistant bacteria in France from the median incidence of invasive infections observed in all EU/EEA countries. The percentage of resistance in species from the EARS-Net data was then applied (ECDC, unpublished data). Our estimates are therefore more precise because they are based on actual national surveillance and hospital data.

Finally, the estimates of the number of deaths from the European study cannot be compared to our results because the associated mortality proportion used in our study differs for some MDRB.

The 2013 CDC report [3] used surveillance data collected between 2009 and 2011 to estimate that there were more than 2 million infections due to antibiotic-resistant microorganisms that year in the United States. The same limitations raised for the European study apply when comparing the CDC results with ours, because the US considered more bacteria-antibiotic combinations than our study did, e.g. infections with *Salmonella*, *Shigella*, tuberculosis, and even *Candida*. The CDC also used a different methodology to calculate the number of deaths, which limits the comparisons still further. It applied the same proportion of deaths — 6.5% — to infections with carbapenemase-producing *enterobacteriaceae*, MDR *Acinetobacter,* ESBL-producing *enterobacteriaceae*, GRE, and MDR *P. aeruginosa*. This figure, derived from a study published in 2009 [26], is an estimate of the overall mortality associated with nosocomial infections due to MDRB. Instead, we used MDR-specific and site-specific mortality ratios for each type of infection, which appears more appropriate, in view of the great variability of associated mortality rates between different infections due to MDRB and body sites.

The specifications of the European study, the CDC American study and our Santé publique France study have been summarised in three additional tables (Additional file 1: Tables a, b, c). The differences in these studies underline the need to define a standardised protocol for international assessments of the morbidity and mortality of antibiotic resistance. Only results from studies based on such a protocol, which could be promoted for example by the ECDC or WHO as part of the global action plan on antimicrobial resistance recently adopted by the World Health Assembly [1], will allow valid comparisons between countries.

We decided here to assess the overall mortality associated with infections due to MDRB and not specifically that associated with antibiotic resistance. Several publications have already reported the excess mortality associated with resistance alone. A study of two matched cohorts of patients [27] showed that the 30-day mortality associated with 3GC-R *E. coli* bloodstream infections was 2.5 times greater (confidence interval: 0.9-6.8) than that associated with susceptible strains. A meta-analysis of 16 studies [28] also showed a significant increased risk in the mortality associated with bloodstream infections due to ESBL-producing *Enterobacteriaceae*, almost twice as high as that observed for susceptible *Enterobacteriaceae* (pooled RR: 1.85; confidence interval: 1.39-2.47).

Several studies suggest that infections due to MDRB do not replace those with susceptible bacteria, but add to them (the so-called “Boyce effect”), thus increasing the total morbidity and mortality of infection from a given bacterium. This characteristic was initially described for MRSA [29], and more recently for 3GC-R *E. coli* [30], two bacteria that account for two thirds of the cases of infections due to MDRB and more than one third of the deaths estimated in our study. A substantial fraction — and if the Boyce effect also applies to other MDRB, the majority — of the deaths we estimate should therefore be considered as excess mortality compared with those associated with infections by susceptible bacteria.

Our estimates are accompanied by uncertainty. To overcome this limit, for morbidity estimates, each PPS-based ratio was assigned a lower and an upper plausibility value, provided by the literature. The number of deaths was calculated by multiplying this ratio with the value of the morbidity estimates and their plausibility intervals, allowing presenting a plausibility interval for mortality estimates too. The numbers of deaths and plausibility intervals for deaths were estimated with these morbidity estimates and their plausibility intervals multiplied with mortality ratio provided by the literature. Part of uncertainty on the mortality estimates is taken into account through these plausibility intervals. However, uncertainty around proportions of deaths remain because of lack of sufficient-sized studies leaded in Europe on MDRB infections mortality.

## Conclusions

The results of our study confirm that multidrug-resistance to antibiotics is a major public health problem in France. The morbidity and mortality associated with infections due to MDRB is particularly important for MRSA and 3GC-R *Enterobacteriaceae*. Surveillance of resistance to these bacteria therefore remains an important priority. The number of infections due to emerging highly resistant bacteria is still limited in France, probably due to the measures implemented to prevent cross-transmission.

The number of cases of infections and the number of deaths associated with infections due to MDRB calculated in this study are indicators that should facilitate communication and awareness of both healthcare professionals and the general public. Repeat this study in a few years with data from the next French PPS study, which will occur in 2017, and EARS-Net data, would provide trends estimations.

Additional studies remain to be conducted, for example to assess the projected morbidity and mortality of antibiotic resistance in France according to several scenarios based on incidence rates and efficacy of control programmes. It is equally important to assess the economic costs (of medical care, of measures to control dissemination in hospitals, and to the community) of infections due to MDRB in order to reinforce the adherence of healthcare professionals and policy-makers to prevention programmes.

## Additional file

Additional file 1: Tables **a**, **b**, **c.** Table a, table b, table c have been added as supplementary material. **Table a** presents the methodology, parameters and main results of 3 studies estimating the morbidity and mortality of multidrug-resistant bacteria infections : the European study, the US study and our French study. **Table b** compares the ratios used for estimating the incidence of non-invasive infections in the European study and Santé publique France study. **Table c** compares the ratios used for estimating mortality associated with infections due to MDRB in the European study and Santé publique France study [31–34]. (PDF 285 kb)

## Abbreviations

3GC-R *E. coli*: *Escherichia coli* resistant to third-generation cephalosporins
3GC-R *K. pneumoniae*: *Klebsiella pneumoniae* resistant to third-generation cephalosporins
3GC-R: Third-generation cephalosporin-resistant
CDC: US Centers for disease control and prevention
CR Acinetobacter spp: Acinetobacter spp. resistant to carbapenems
CR *K. pneumoniae*: K. pneumoniae resistant to carbapenems
CR P. aeruginosa: Pseudomonas aeruginosa resistant to carbapenems
EARS-Net: European antimicrobial resistance surveillance network
ECDC: European centre for disease prevention and control
ESBL: Extended-spectrum beta-lactamase producing enterobacteriaceae
EU/EEA countries: European union and European economic area countries
GRE: *Enterococcus faecium* and *E. faecalis* resistant to glycopeptides
HD: Inpatient hospital days
MDR: Multidrug resistant
MDRB: Multidrug resistant bacteria
MRSA: Methicillin-resistant *Staphylococcus aureus*
PPS 2012: Point prevalence survey of healthcare-associated infections and antimicrobial use in French hospitals in 2012
