# Supplementary material for: Estimating the morbidity and mortality associated with infections due to multidrug-resistant bacteria (MDRB), France, 2012
Source: Antimicrob Resist Infect Control. 2016 Dec 12;5:56. doi: 10.1186/s13756-016-0154-z (PMC5154061; doi:10.1186/s13756-016-0154-z)
Supplement: Supplementary file 1 — a, b, c. Table a, table b, table c have been added as supplementary material. Table a presents the methodology, parameters and main results of 3 studies estimating the morbidity and mortality of multidrug-resistant bacteria infections : the European study, the US study and our French study. Table b compares the ratios used for estimating the incidence of non-invasive infections in the European study and Santé publique France study. Table c compares the ratios used for estimating mortality associated with infections due to MDRB in the European study and Santé publique France study [31–34]. (PDF 285 kb) [file 13756_2016_154_MOESM1_ESM.pdf]

Table a – Parameters used in 3 studies estimating the morbidity and mortality of multidrug-resistant bacteria infections

Study	ECDC/EMEA study [2]	Santé publique France study	CDC study [3]
**Geographic area concerned**	Europe	France	USA
**Criteria for MDRB selection**	- being frequently responsible for bloodstream infections<br>- AND the associated antibiotic resistance is, in most cases, a marker for multiple resistance to antibiotics<br>- AND data available in the European Antimicrobial Resistance Surveillance Network (EARS-Net)	· being associated with invasive infections, i.e., infections with a bacteria isolated from blood or cerebrospinal fluid<br>· AND having a significant prevalence or having emerged recently<br>· AND being included in a surveillance network in France<br>· AND being multidrug-resistant as defined in the EARS-Net protocol	- bacteria causing severe human infections, resistant to the antibiotics used to treat those infections<br>- for *Candida* : showing increasing resistance to the drugs used for treatment
**MDRB selected**			
MRSA[a]	YES	YES	YES
VISA/VRSA[b]	-	-	YES
S. pneumoniae	YES (penicillin resistant) for morbidity estimations only	-	YES (fully resistant to clinically relevant drugs)
GRE[c]	YES (*E. faecium* only)	YES (*E. faecium* + *E. faecalis*)	YES
3GC-R[d] *E. coli*	YES	YES	YES (ESBL[e] producing only)
CR[f] *E. coli*	-	-	YES
3GC-R *K. pneumoniae*	YES	YES	ESBL producing only
CR *K. pneumoniae*	-	YES	YES (ESBL[e] producing only)
CR[g] *P. aeruginosa*	YES	YES	YES (resistant to 3 or more drug classes)
Acinetobacter spp	-	YES (resistant to imipenem or meropenem, or doripenem)	YES (resistant to 3 or more drug classes)
Other	-	-	YES*
**Data used for morbidity estimations**	EARS-Net data (2007) corrected with a pathogen specific rate for keeping nosocomial infection only (eg : 65% for MRSA; ECDC data not shown)	EARS-Net data (2012)	· for ESBL-producing and CR *E. coli*, ESBL-producing and CR *K. pneumoniae*, multidrug-resistant *Acinetobacter spp*, GRE and multidrug-resistant *P. aeruginosa* :<br>○ 2011 survey of 11,282 patients for proportion of pathogen-specific HAI[h]<br>○ estimated for USA using 2010
**Method for extrapolation to the geographic area concerned**	Population covered by EARS-Net for each type of bacteria was obtained directly from countries of the EARS network, except for France : the proportion of resistance reported for	Population covered by EARS-Net was estimated from the number of inpatient hospital days reported by hospitals whose laboratories had transmitted data to the network (secondary and	

	France in EARS-Net was applied to the median incidence of nosocomial bloodstream infections observed in all European countries (ECDC data not shown).	tertiary care hospitals)	Nationwide Inpatient Sample<br>○ corrected by CDC's National Healthcare Safety Network data 2009-2010 for proportion of non-susceptible pathogens<br>· for MRSA : Emerging Infection Program/Active Bacterial Core Surveillance (ABC) data 2011 extrapolate to USA using 2011 data from National Center for Health Statistics and US renal data Systems<br>· for VRSA : individual case reports confirmed by CDC (nationally notifiable condition)<br>· for *S. pneumoniae* : rate for full resistance to clinically relevant drugs in 2011 in ABC (30%) applied to estimates of cases of all *S. pneumoniae* infections as estimated in a previous study [31]
**Infections included**	Bloodstream infections, lower respiratory tract infections, skin and soft tissue infections and urinary tract infections	- for all MDRB: invasive infections (bacteria isolated from blood or cerebrospinal fluid), urinary tract infections, skin and soft tissue infections, and surgical site infections ;<br>- for all MDRB excluding GRE: respiratory tract infections ;<br>- for MRSA only: bone and joint infections (does not include surgical site infections);<br>- for GRE only: gastrointestinal tract infections (gastro-intestinal and intra-abdominal infections).	all infections, except for MRSA (invasive infection only)
**Origin of infection**	Nosocomial infection only, except for *S. pneumoniae*	Healthcare and community associated infections, but diagnosed in a secondary or tertiary hospital.	HAIs infections with onset in hospitalized patients only, except for MRSA, VRSA and *S. pneumoniae* : healthcare and community associated.
**Ratio used for estimating non-invasive infections**	Specific morbidity ratio for each MDRB and each body site, from literature (table b)	Three specific morbidity ratios for each MDRB and each body site, from literature and from the French Point prevalence Study 2012 [7] in order to have a target value and intervals of plausibility (table b)	Not applicable

**Ratio used for mortality**	Specific mortality ratio for each MDRB and each body site (table c)	Specific mortality ratio for each MDRB and each body site (table c)	· for ESBL-producing and CR *E. coli*, ESBL-producing and CR *K. pneumoniae*, multidrug-resistant *Acinetobacter spp*, GRE and multidrug-resistant *P. aeruginosa* : 6,5% (overall estimate of attributable mortality from antibiotic-resistant hospital onset infections as determined in a previous study [26])<br>· For MRSA : mortality from all causes during hospitalization, extrapolate to USA using 2011 data from National Center for Health Statistics and US renal data Systems<br>· For VRSA : Not applicable (too few infections)<br>· For *S. pneumoniae* : rate of full resistance applied to the total number of death from pneumococcal disease as estimated in a previous study [31]
**Main results**			
Morbidity estimation	386,100 cases	158,000 (127,000 to 245,000) cases	2,049,442 cases per year
Mortality estimation	25,100 extra deaths	12,500 (11,500 to 17,500) deaths	23,000 deaths per year

[a]Methicillin resistant *Staphylococcus aureus*
[b]Vancomycin-intermediate resistant and resistant *Staphylococcus aureus*
[c]Glycopeptid resistant *Enterococcus*
[d]Third-generation cephalosporin resistant : resistance to cefotaxime or ceftriaxone or ceftazidime
[e]Extended-spectrum beta-lactamase
[f]Carbapenem resistant : resistance to imipenem or meropenem
[g]Carbapenem resistant : imipenem, meropenem, doripenem
[h]Healthcare-associated infections
*Drug-resistant *Neisseria gonorrhoeae* (any drug), Drug-resistant *Campylobacter,* Fluconazole-resistant *Candida,* Drug-resistant Non-typhoidal *Salmonella,* Drug-resistant *Salmonella Typhi*, Drug-resistant *Shigella,* Drug-resistant tuberculosis, Erythromycin-resistant Group A *Streptococcus,* Clindamycin-resistant Group B *Streptococcus*

Table b - Ratios used for estimating the incidence of non-invasive infections, ECDC/EMEA vs. Santé publique France studies.

Body sites	ECDC/EMEA study [2]	Santé publique France Study		
		PPS ratio	Ratios from the literature	
			Low value	High value
No. MRSA urinary tract infections/No. MRSA invasive infections	0.75 [11]	0.98 [7]	0.75 [11]	2.30 [12]
No. MRSA respiratory infections/No. MRSA invasive infections	1.25 [11]	2.37 [7]	1.25 [11]	2.50 [12]
No. MRSA skin and soft tissue infections and surgical site infections/No. MRSA invasive infections	5.25 [11]	4.17 [7]	4.90 [12]	5.25 [11]
No. MRSA bone and joint infections/No. MRSA invasive infections	-	0.79 [7]	[b]	1.38 [12]
No. GRE urinary tract infections/No. GRE invasive infections	3.44 [14]	4.33 [7]	2.33 [13]	3.44 [14]
No. GRE gastrointestinal tract infections /No. GRE invasive infections	1.89 [14]	2.65 [7]	1.89 [14]	[b]
No. GRE skin and soft tissue infections and surgical site infections/No. GRE invasive infections	4.67 [14]	1.46 [7]	4.67 [14]	5.00 [13]
No. penicillin resistant *S. pneumoniae* respiratory infections/No. invasive infections	2,7 [32]	-	-	-
No. 3GC-R *E. coli* urinary tract infections/No. 3GC-R *E. coli* invasive infections	1.19 [15]	5.50 [7]	2.30 [13]	[b]
No. 3GC-R *E. coli* respiratory tract infections/No. 3GC-R *E. coli* invasive infections	1.19 [15]	1.08 [7]	[b]	2.50 [13]
No. 3GC-R *E. coli* skin and soft tissue infections and surgical site infections/No. 3GC-R *E. coli* invasive infections	0.33 [15]	1.37 [7]	[b]	4.90 [13]
No. 3GC-R *K. pneumoniae* urinary tract infections/No. 3GC-R *K. pneumoniae* invasive infections	1.19 [15]	2.90 [7]	1.19 [15]	2.30 [13]
No. 3GC-R *K. pneumoniae* respiratory tract infections/No. 3GC-R *K. pneumoniae* invasive infections	1.19 [15]	2.55 [7]	1.19 [15]	2.50 [13]
No. 3GC-R *K. pneumoniae* skin and soft tissue infections and surgical site infections/No. 3GC-R *K. pneumoniae* invasive infections	0.33 [15]	0.82 [7]	0.33 [15]	4.90 [13]
No. CR *K. pneumoniae* urinary tract infections/No. CR *K. pneumoniae* invasive infections	-	2.71 [7]	3.00 [16]	3.10 [17]
No. CR *K. pneumoniae* respiratory tract infections/No. CR *K. pneumoniae* invasive infections	-	3.10 [7]	0.42 [17]	1.60 [16]
No. CR *K. pneumoniae* skin and soft tissue infections and surgical site infections/No. CR *K. pneumoniae* invasive infections	-	0.44[a]	0.28 [17]	0.60 [16]
No. CR *P. aeruginosa* urinary tract infections/No. CR *P. aeruginosa* invasive infections	11.30 [15]	2.11 [7]	[b]	11.30 [15]
No. CR *P. aeruginosa* respiratory tract infections/No. CR *P. aeruginosa* invasive infections	16.00 [15]	10.99 [7]	[b]	16.00 [15]
No. CR *P. aeruginosa* skin and soft tissue infections and surgical site infections/No. CR *P. aeruginosa* invasive infections	4.67 [15]	3.07 [7]	[b]	4.67 [15]
No. CR *Acinetobacter* urinary tract infections/No. CR *Acinetobacter* invasive infections	-	0.58[a]	0.32 [18]	0.83 [17]
No. CR *Acinetobacter* respiratory tract infections/No. CR *Acinetobacter* invasive infections	-	4.64 [7]	1.21 [18]	3.83 [17]
No. CR *Acinetobacter* skin and soft tissue infections and surgical site infections/No. CR *Acinetobacter* invasive infections	-	0.73[a]	0.29 [18]	1.17 [17]

[a] *no 2012 PPS data for these infections because the prevalence of these MDRB is too low in France; the ratio value applied is the mean value of the 2 ratios from the literature*

[b] *no data in the literature for these infections; the value of the 2012 PPS ratio was applied*

Invasive infections: bacteria isolated from blood or cerebrospinal fluid

Table c – Ratios used for estimating mortality associated with infections due to MDRB, ECDC/EMEA vs. Santé publique France studies.

Body sites	Associated deaths (%)	
	**ECDC/EMEA study [2]**	**Santé publique France study**
MRSA invasive infections	9.8 [19]	9.8 [19]
MRSA urinary tract infections	0.2 [10, 19]	0.2 [10, 19]
MRSA respiratory tract infections	7.0 [10, 19]	7.0 [10, 19]
MRSA skin and soft tissue infections and surgical site infections	1.4 [10, 19]	1.4 [10, 19]
MRSA bone and joint infections	-	9.8[d]
GRE invasive infections	25.0 [14]	25.0 [14]
GRE urinary tract infections	9.0 [14]	9.0 [14]
GRE gastrointestinal tract infections	3.0 [14]	3.0 [14]
GRE skin and soft tissue infections and surgical site infections	6.0 [14]	6.0 [14]
3GC-R *E. coli* invasive infections	30 [33]	18.0 [20]
3GC-R *E. coli* urinary tract infections	1 [10, 33]	0.0 [21]
3GC-R *E. coli* respiratory tract infections	21 [10, 33]	12.9 [10, 20]
3GC-R *E. coli* skin and soft tissue infections and surgical site infections	4 [10, 33]	2.6 [10, 20]
3GC-R *K. pneumoniae* invasive infections	30 [33]	18.0 [20]
3GC-R *K. pneumoniae* urinary tract infections	1 [10, 33]	0.4 [10, 20]
3GC-R *K. pneumoniae* respiratory tract infections	21 [10, 33]	12.9 [10, 20]
3GC-R *K. pneumoniae* skin and soft tissue infections and surgical site infections	4 [10, 33]	2.6 [10, 20]
CR *K. pneumoniae* invasive infections	-	37.0 [22]
CR *K. pneumoniae* urinary tract infections	-	0.8 [10, 22]
CR *K. pneumoniae* respiratory tract infections	-	26.4 [10, 22]
3GC-R *K. pneumoniae* skin and soft tissue infections and surgical site infections	-	5.4 [10, 22]
CR *P. aeruginosa* invasive infections	17.7 [34]	33.0 [23]
CR *P. aeruginosa* urinary tract infections	0.4 [10, 34]	0.8 [10, 23]
CR *P. aeruginosa* respiratory infections	12.7 [10, 34]	23.6 [10, 23]
CR *P. aeruginosa* skin and soft tissue infections and surgical site infections	2.6 [10, 34]	4.8 [10, 23]
CR *Acinetobacter* invasive infections	-	36.5 [24]
CR *Acinetobacter* urinary tract infections	-	0.8 [10, 24]
CR *Acinetobacter* respiratory infections	-	26.1 [10, 24]
CR *Acinetobacter* skin and soft tissue infections and surgical site infections	-	5.3 [10, 24]

[d] *no data could be found in literature, the fraction applied is therefore that estimated for invasive infections.*
Invasive infections: bacteria isolated from blood or cerebrospinal fluid
